# Varus morphology and its surgical implication in osteoarthritic knee and total knee arthroplasty

**DOI:** 10.1186/s13018-022-03184-4

**Published:** 2022-06-03

**Authors:** Chiara Suardi, Davide Stimolo, Luigi Zanna, Christian Carulli, Matassi Fabrizio, Roberto Civinini, Matteo Innocenti

**Affiliations:** Orthopaedic Clinic CTO, University of Florence, Largo Palagi 1, 50139 Florence, Italy

**Keywords:** Varus knee, Total knee arthroplasty, Lower limb alignment, Medial proximal tibial angle

## Abstract

**Background:**

Knee varus alignment represents a notorious cause of knee osteoarthritis. It can be caused by tibial deformity, combined tibial–femoral deformity and/or ligament imbalance. Understanding malalignment is crucial in total knee arthroplasty to restore frontal plane neutral mechanical axis. The aim of this study was to determine which factor contributes the most to varus osteoarthritic knee and its related surgical implications in performing a total knee arthroplasty.

**Methods:**

We retrospectively evaluated 140 patients operated for total knee arthroplasty due to a varus knee. Full-leg hip to ankle preoperative X-rays were taken. Radiological parameters recorded were: mechanical axis deviation, hip–knee–ankle, anatomical–mechanical angle, medial neck shaft angle, mechanical lateral distal femoral angle (mLDFA), medial proximal tibial angle (MPTA), joint line convergence angle (JLCA), lateral proximal femoral angle, lateral distal tibial angle (LDTA), femoral bowing, and length of tibia and femur. We also determined ideals tibial and femoral cuts in mm according to mechanical alignment technique. A R2 was calculated based on the linear regression between the predicted values and the observed data.

**Results:**

The greatest contributor to arthritic varus (*R* = 0.444) was MPTA. Minor contributors were mLDFA (*R* = 0.076), JLCA (*R* = 0.1554), LDTA (*R* = 0.065), and femoral bowing (*R* = 0.049). We recorded an average of 7.6 mm in lateral tibial cut thickness to restore neutral alignment.

**Conclusions:**

The radiological major contributor to osteoarthritic varus knee alignment is related to proximal tibia deformity. As a surgical consequence, during performing total knee arthroplasty, the majority of the correction should therefore be made on tibial cut.

## Introduction

Varus malalignment represents a notorious cause of knee osteoarthritis in adults. The pathogenesis is correlated with an increased loading of the medial tibiofemoral compartment that can lead to faster degeneration of the cartilage of medial compartment. Varus malalignment of the knee may be caused by tibial deformity, combined tibial and femoral deformity, and may also present ligament imbalance [1–3]. This malalignment can occur constitutionally, which refers to a knee with a HKA (hip–knee–ankle angle) < 3°, or secondary to injuries, tumors, and other pathologies [1, 2, 4, 5]. It is already well known in the literature that medial proximal tibial angle (MPTA) contributes the most to a varus knee in case of varus constitutional conformation [6, 7]. Conversely, regarding varus knee deformity for all causes but constitutional varus, the literature offers great variability of observations [7–12]. Indeed, some authors pointed the mechanical lateral distal femoral angle (mLDFA) and the medial neck shaft angle (MNSA) as important contributors to varus [7, 8]. Others also observed that the lateral femoral bowing could be related to progression of varus osteoarthritic knee [9].

In total knee arthroplasty (TKA), preoperative planning is mandatory to establish the limb alignment (varus or valgus) and to determine the eventual correctability of knee deformities [13]. Indeed, preoperative planning allows to estimate soft tissue status and ligament balance.

Moreover, there is great interest to understand and classify knee deformity not only based on HKA but also on the coronal joint line orientation. As demonstrated in different studies [14–16], the joint line obliquity can be determined by different combination of femoral and tibial phenotypes, both in varus and in valgus knees. The knowledge of the individual role of these patterns could be the key to personalize the reconstructive procedures in the future.

The aim of this study was to determine which factor contributes the most to varus osteoarthritic knee and which are the clinical implications in performing a TKA in a varus knee [6, 7]. The hypothesis was that varus osteoarthritic knee is related to many lower limb parameters some of that related to the constitutional varus and other to the osteoarthritis disease progression itself.

## Materials and methods

We retrospectively evaluated 140 patients operated for TKA due to a varus knee between 2016 and 2018 at our Institution. There were 106 female and 34 male patients. Inclusion criteria to the study group consisted of all patients of any age or gender with a weight-bearing full-leg preoperative X-rays on a bipodal stance showing varus mechanical axis alignment.

### Radiological assessment

**Fig. 1:**
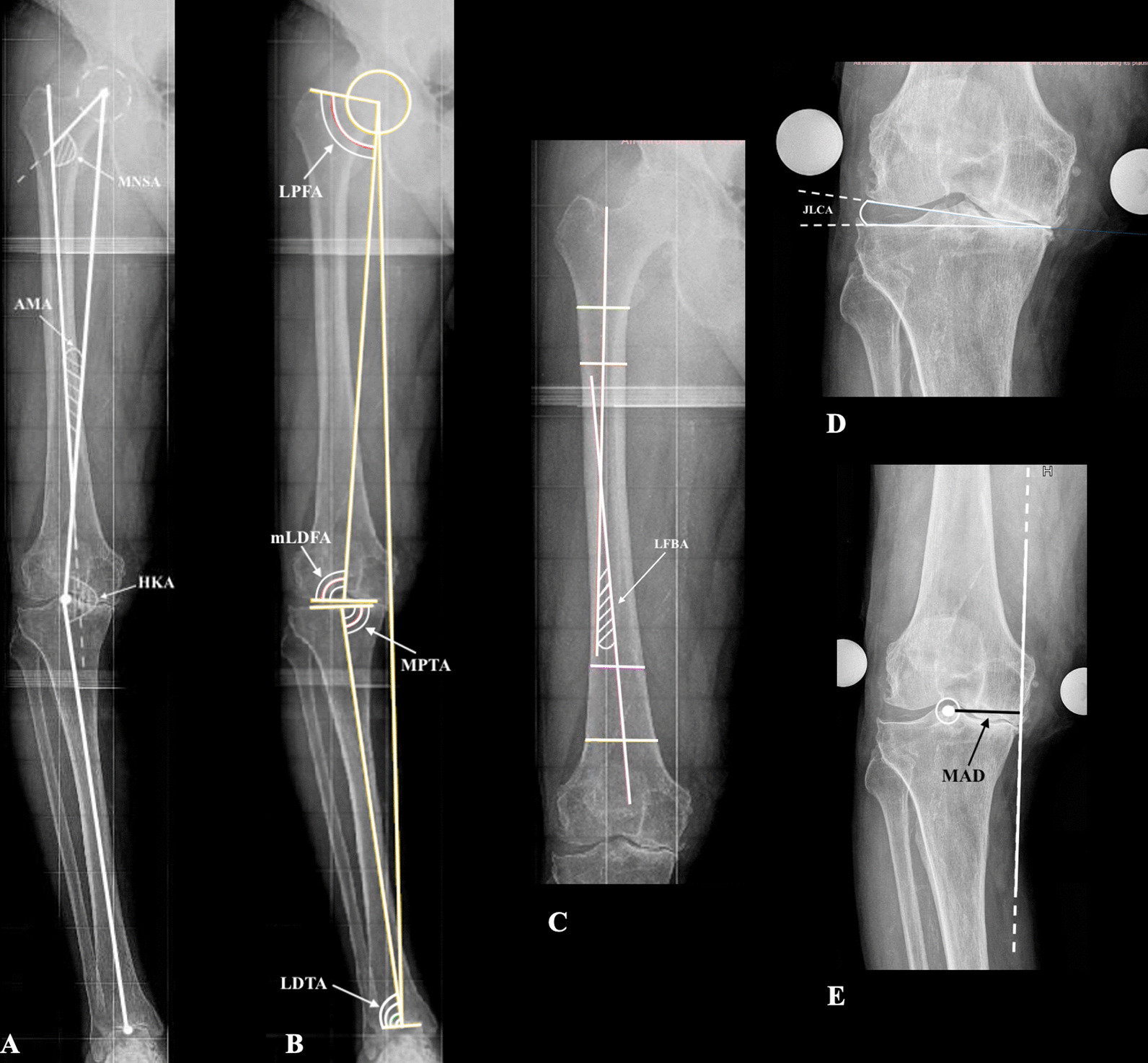
Radiographic assessment of preoperative measurements. **A** *HKA* hip–knee–ankle angle, *AMA* anatomical–mechanical angle, *MNSA* medial neck shaft angle. **B** Deformity analysis angles. *LPFA* lateral proximal femoral angle, *mLDFA* mechanical lateral distal femoral angle, *MPTA* medial proximal tibial angle, *LDTA* lateral distal tibial angle. **C** *LFBA* lateral femoral bowing angle. **D** *JLCA* joint line converge angle. **E** *MAD* mechanical axis deviation distance

One hundred and forty TKAs were preoperatively classified as varus aligned in our database by the measurement of HKA, according to what has been postulated by Paley [2] and recently revised by Bahadir et al. [17]. In the neutrally aligned limb, the HKA angle approaches 180°. (Varus deviations are negative HKA angle and valgus deviations are positive HKA angle.) The weight-bearing full-leg radiographs were obtained as described by Paley [18] with the subjects standing barefoot and the feet together at attention position, while the patellae were oriented forward. This standard position ensured that the tibias were vertical and facing forward with minimal rotation. Two observers performed all the X-ray measurements independently from each other to power up the accuracy of the investigation. We used Carestream Health (Rochester, NY) for all analyses. The center of the femoral head was determined using a digital template with concentric circles. The center of the knee was determined as the intersection of the midline between the tibial spines and the midline between the femoral condyles and tip of the tibiae. The center of the ankle was determined as the middle of the talus. The mechanical femoral axis was defined as the line from the center of the femoral head to the center of the knee. The mechanical tibial axis was defined as the line from the center of the knee to the center of the ankle. The anatomical femoral axis was defined as the line from the center of the knee to the bisector of the medullary canal of the femur. For length measurements, the distance from the center of the femoral head to the center of the knee was defined as the femoral length. We evaluated radiologic parameters as described first by Paley [2] and then revised by Bahadir et al. [17] including the mechanical axis deviation (MAD), hip–knee–ankle (HKA), anatomical–mechanical angle (AMA), medial neck shaft angle (MNSA), mechanical lateral distal femoral angle (mLDFA), medial proximal tibial angle (MPTA), joint line convergence angle (JLCA), lateral proximal femoral angle (LPFA), lateral distal tibial angle (LDTA), femoral bowing, and length of tibia and femur (Fig. 1A–E). MAD is the distance between the mechanical axis line and the center of the knee. Medial MAD was referred to as varus alignment, while lateral MAD was referred to as valgus alignment. HKA was defined as the angle formed by the mechanical femoral axis and the mechanical tibial axis. The HKA value included between 178° and 182° was defined as normal alignment, as varus with a negative value and as valgus with positive value. AMA was defined as the angle between the anatomical and mechanical femoral axes. MNSA was defined as the angle between the longitudinal axis of the neck and the longitudinal axis of the femoral shaft. mLDFA was defined as the lateral angle formed between the mechanical femoral axis and the knee joint line of the distal femur. The MPTA was defined as the medial angle formed between the mechanical tibial axis and the knee joint line of the proximal tibia. The JLCA was defined as between the tangent through the two most convex distal points of the femoral condyles and a line along the flat portion of the subchondral bone of the tibial plateau. The LPFA was defined as the angle between the line connecting the tip of the greater trochanter with the center of the femoral head and the mechanical femoral axis. The LDTA was defined as the angle between the mechanical tibial axis and a line through the tip of the medial and lateral talus shoulder. Lateral femoral bowing was defined as an acute angle formed between the line drawn at the center of the femur below the level of the lesser trochanter to pass the center of the femur at a point 5 cm distal to the starting point and the line extending from the center of the femoral distal condyle through the center of the femur at a 5 cm proximal portion and a 5 cm further proximal point (Fig. 1A–E). In the presence of a JLCA > 2°, associated with a clinical positive varus stress at 0° and/or 30° of flexion, a stress radiograph was made to evaluate the varus stress JLCA. This angle was only checked preoperatively to determine the joint line congruency under stress and so to evaluate the lateral soft tissues straining. All other measures were performed pre- and postoperatively at 6 weeks after surgery. On preoperative calibrated digital radiographs, we planned the thickness of tibial and femoral cuts in millimeters following the principles of mechanical alignment, making resections at 90° to the femoral and tibial mechanical axes. Intraoperatively, with the use of a caliber, we measured and recorded the real thickness of the resected medial–lateral tibial plates bone cuts and medial–lateral distal femoral condyles bone cuts. The two measurements, the ones based on preoperative planning and the ones measured during surgery, were then compared.

### Statistical analysis

We used the software StatPlus:mac for Macintosh to elaborate statistical data. We analyzed the correlation between HKA median value and all other recorded median parameters. Every observation was made by the regression linear model using the ordinary least squares. An R2 was calculated based on the linear regression between the predicted values and the observed data. An *R* proximal to 1 was considered a good predictor of the dependent variable, while an *R* proximal to 0 represents the absence of correlation and -1 represents a negative correlation. We put all data on the *x*–*y* graphics for each parameter to calculate the straight-line equation between all HKA values and all other parameters. This line estimates the nature of data correlation between HKA and all other parameters (directly vs inversely proportional). The “intraclass correlation coefficient” (ICC) was used to measure the variability in between the digitally planned cuts’ thickness and the effective cuts performed intraoperatively. The ICC was also performed among different observers. An ICC between 0.75 and 1.00 was considered as excellent (almost no variability in between the two measurements).

## Results

**Fig. 2:**
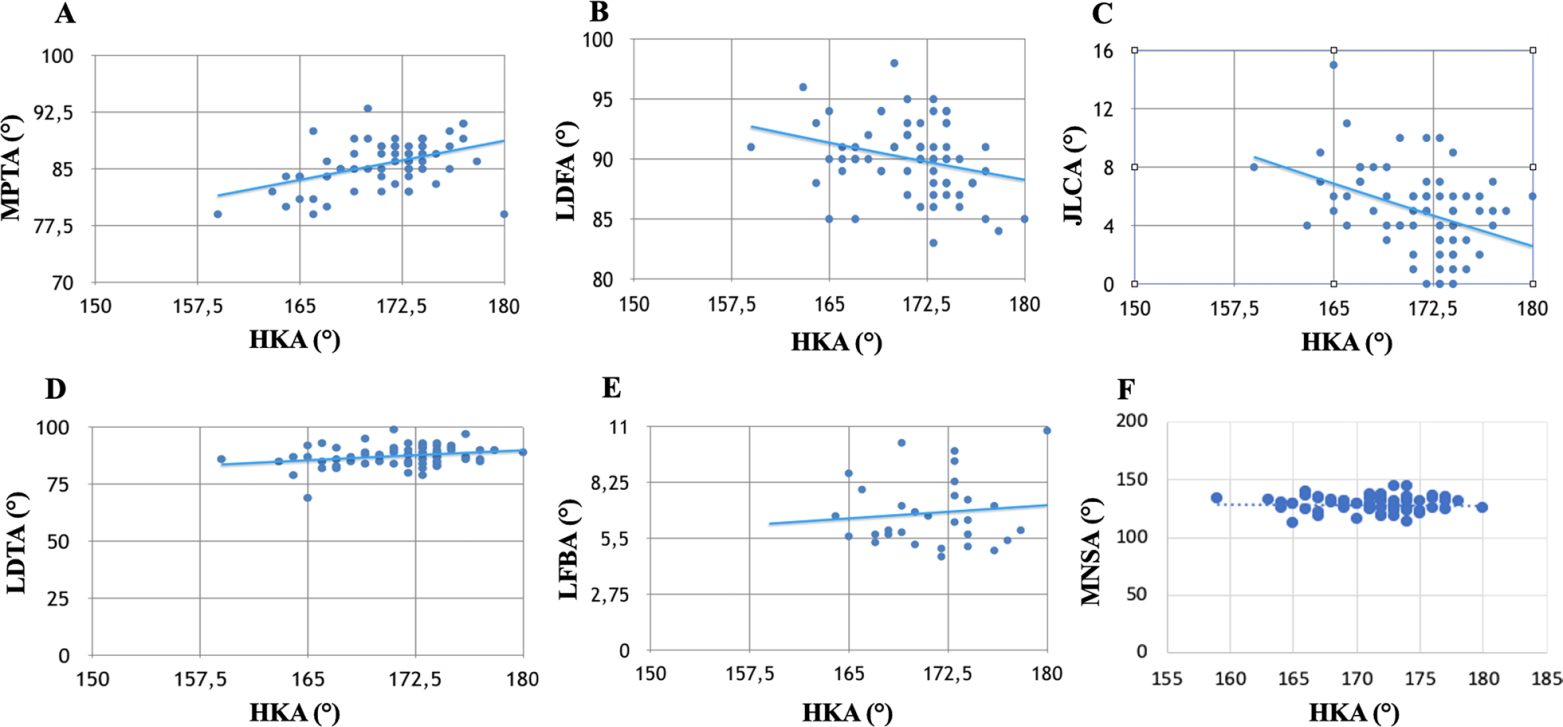
Linear regression between all preoperative angle assessments and HKA angle. All values are expressed in degrees. **A** Linear regression between MPTA and HKA. **B** Linear regression between LDFA and HKA. **C** Linear regression between JLCA and HKA. **D** Linear regression between LDTA and HKA. **E** Linear regression between femoral bowing and HKA. **F** Linear regression between MNSA and HKA

There were 106 females (75%) and 34 males (24%). Everyone had varus alignment with a HKA between 159° and 174° (average of 171°). According to Bahadir et al. [17], 92 knees (65%) had an osseous malalignment that is the presence of a tibial varus deformity [MPTA < 85° (median 85.7°)], or femoral varus deformity (LDFA > 90° (median 90°)) or combined femoral and tibial varus deformity (LDFA > 90° and MPTA < 85°). Forty-four (31%) of these had a tibia vara deformity, 30 (21%) a femoral varus deformity, and 12 (8.5%) a combined femoral and tibial varus deformity. In 48 knees (34%), we identified an intra-articular malalignment, that is the presence of normal femur and tibia osseous alignment with an altered knee joint congruity: JCLA > 2° (median 5°). Six of them had an associated clinical positive varus stress; therefore, we performed stress radiographs that showed a mean varus stress JLCA of 5.1 ± 1.1. Ten knees (7%) had a combination of osseous malalignment and JLCA > 2° (Table 1). The greatest contributor to arthritic varus (*R* = 0.444) was MPTA. JLCA was the second more relevant parameter correlated with varus deformity even if associated with low *R* (*R* = 0.1554). LDTA (*R* = 0.065), femoral bowing (*R* = 0.049), and MNSA (*R* = 0.003) showed very low association with varus deformity (Fig. 2A–F).

**Table 1:** Radiographic assessments and intraoperative findings

Parameter	Range values	Median
HKA angle (°)	159–180	171.3
mLDFA (°)	83–98	90
MPTA (°)	79–93	85.7
JLCA (°)	0–15	5
MNSA (°)	113–145	128.7
LPFA (°)	70–103	88.8
LDTA (°)	69–99	87
Femoral bowing angle (°)	4.6–7.8	6.7
MAD (mm)	3–76	32.5
Distal medial femoral cut (mm)	3.1–14.7	7.2
Distal lateral femoral cut (mm)	1.8–12.5	6.8
Medial tibial plateau cut (mm)	1.7–13.2	6.5
Lateral tibial plateau cut (mm)	2.2–20.5	7.6
AMA (°)	4–8	6.2
Femoral length (mm)	393–791	466.5
Tibial length (mm)	282–417	354.4

**Fig. 3:**
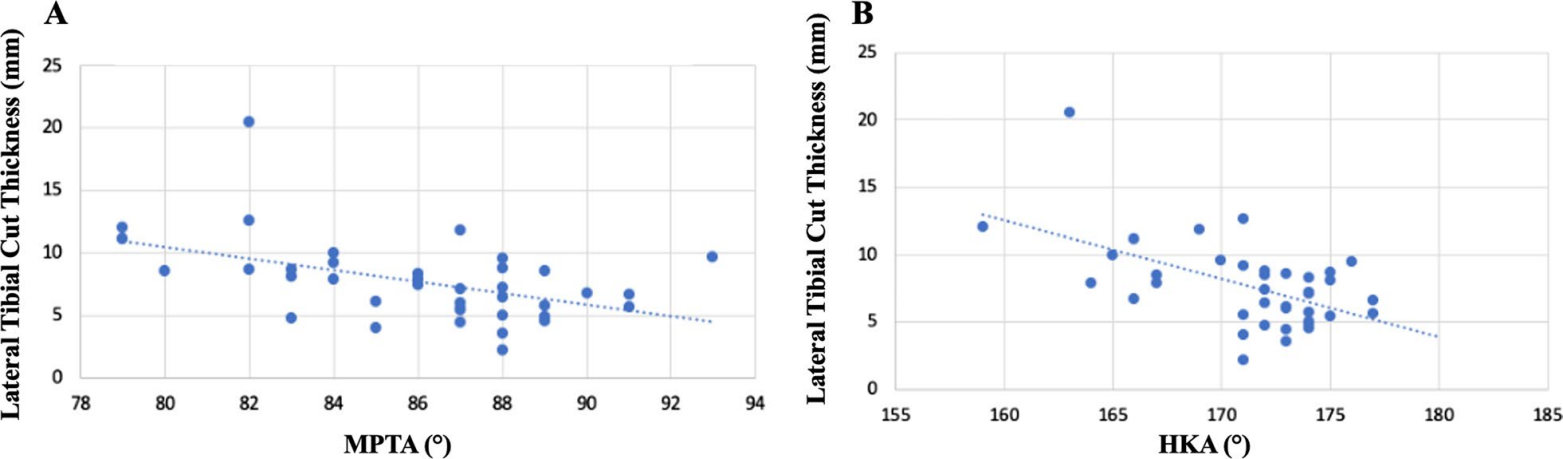
Linear regression between lateral tibial cut thickness and both MPTA (**A**) and HKA (**B**)

We found an average of 7.6 mm of thickness of the lateral tibial cut (range 2.2–20.5 mm). An ICC of 0.92 was found in between the preoperative planned cuts’ thickness and the intraoperative cuts’ thickness. Lateral tibial cut showed direct correlation with an increase in cuts’ thickness and a reduction in both MPTA and HKA values (*R* = 0.471 and *R* = 0.543, respectively) (Table 1). Indeed, in most of the cases, the thickness of the lateral tibial cut was inversely related to the degree of both MPTA and HKA (Fig. 3A, B). The mean HKA was corrected from a preoperative of 170° (SD ± 4) to postoperative 180° (SD ± 2).

The ICC (95% CI) among observers showed an absolute agreement between raters: Interrater reliability between measurements was 0.96.

## Discussion

Meniscal damage, meniscal extrusion, varus–valgus malalignment, and medial–lateral laxity are local factors that may be present in primary knee osteoarthritis [19]. Varus malalignment increases loading of the medial tibiofemoral compartment during gait by increasing the external adduction moments (force toward the sagittal plane) acting on the knee during the late-stance phase of the gait cycle [20, 21]. In TKA, gold standard is restoration of neutral mechanical axis, considered critical for long-term success. A tibial cut made at 90° compared to the tibial mechanical axis allows long survival of the tibial component in terms of aseptic loosening [10–12]. However, in the last 10 years there have been proposed alternative techniques for lower limb alignment [22], and recent data are challenging the superiority of mechanical alignment [23]. The new trend in reconstructive knee surgery is to analyze the coronal deformity of femur and tibia and their role in determining the direction of joint line obliquity, in order to personalize the alignment to pursue [14, 15].

In this study, we aimed to discriminate factors that contribute to arthritic varus and to the global limb malalignment. We observed several of our patients with varus angulation of their tibia, contributing to the global limb malalignment. In the literature, different observations are reported about morphology to varus arthritic knee. Bellemans et al. previously demonstrated that the strongest parameter that influences HKA in healthy knees with constitutional varus is MPTA [7]. Similarly, in our study, the deformity of the proximal tibia was the most important factor related to the varus osteoarthritic knee (Fig. 2A). Varus malalignment determines a medial overload that results in a tibial cartilage wearing with secondary subchondral bone collapse. Correcting intra-operatory by cutting the most lateral tibial plateau with the guide aligned 90° from mechanical axis of the tibia, we observed a restoration of neutral limb alignment. Moreover, tibial cuts measured preoperatively were predictable of what have been measured during surgery.

Weiping et al. [24] observed that femorotibial geometric alignment referred to increased mLDFA and decreased MPTA was one of the two potential components giving the major contribution to varus deformity of the lower extremity in knee osteoarthritis. In our population, the conformation of the distal femur had a minor impact in the contribution of varus knee than the geometry of the proximal tibia. At the same time, observing the correlation diagram between mLDFAs and HKAs, millimetric augment of varus grade on the distal femur corresponds linearly to HKA varus grade augmentation (Fig. 2B). Bellemans [7] observed a good correlation of these two parameters also in constitutional knees.

Thienpont et al. [25] suggest that the mean varus alignment of the lower limb (178° HKA°) is rather a result of lateral soft tissue laxity with joint line opening (JLCA of 3°) on the lateral side in varus knees. Although in our population we found a median JLCA of 5°, it was not significantly related to the amount of varus osteoarthritic deformity as it was for the MPTA. Moreover, the amount of JLCA did not change the definitive tibial plateau cuts’ thickness. Tibial cuts had major thickness on the lateral side than in the medial side. That was directly related to the grade of varus deformity and so on the grade of MPTA (Fig. 3A,B). Secondary to these observations, the majority of correction during TKA should be obtained on the lateral tibial plateau, in order to correct tibial deformity. A JLCA > 2° could be more related to the amount of medial soft tissue release needed to balance the knee in extension once the mechanical alignment of the tibia has already been restored through the tibial cut. This is definitively important in order to get a neutral, well-balanced, HKA. Indeed, not so much intra-articular deformity correction can be obtained on the femoral side compared to the tibial side associated with soft tissue release in extension on the concave side (medial collateral ligament in extension).

Cho et al. [9] in a selected Asiatic population found that lateral femoral bowing shows a tendency to increase his value directly proportional to the grade of varus knee deformity. In our European population, we also found an increase in femoral bowing according to the degree of varus knee deformity (Fig. 2E), but not statistically significant and with mean values at the edge of normal parameters (4.6–7.8°). Therefore, diaphyseal femoral deformity had not such a strong impact both on HKA and on osteoarthritis progression in knee varus deformity, compared to what was found in Asiatic population (Table 1).

Finally, Issin et al. [8] shows that abnormal forces applied to ankle may cause collapse in distal lateral tibial metaphysis and decrease LDTA in varus knees and that medial neck shaft angle may decrease due to possible abnormal loading angles to the femoral neck in some individuals with varus gonarthrosis. In our series, different grades of HKA were associated with variable LDTA values showing a completely dissociated correlation between those parameters (Fig. 2D). The same was observed for MNSA (Fig. 2F).

The limitations to this study include the nature of retrospective analysis. We used standard full-leg standing radiographs, which are the standard for alignment assessment, but may not be as accurate and reproducible as 3D computer tomography or biplanar radiographs. Furthermore, the use of bipodal weight-bearing view without the augment of the unipodal view cannot be helpful to evaluate the presence of ligament imbalance. The rotational position of the lower extremities might influence the outcome of the measurements. We performed varus–valgus stress radiograph only in the case of clinical varus positive stress at 0° and/or 30°. We did not compare the grade of medial compartment release to JLCA values. Another limitation is the lack of explanation for the patho-etiology and the natural course of tibia vara. Furthermore, we do not have consecutive images to document the natural progression of this condition overtime.

According to our findings, the major contributor to osteoarthritic varus knee malalignment on radiological evaluation is related to proximal tibia deformity. As a clinical consequence, performing TKA requests consciousness of lower limb alignment. Preoperative planning could be mandatory to investigate the exact position of deformity. During performing TKA, the majority of the correction should therefore be made on tibial cut.

## Data Availability

Not applicable.

## Abbreviations

TKA: Total knee arthroplasty
MAD: Mechanical axis deviation
HKA: Hip–knee–ankle
AMA: Anatomical–mechanical angle
MNSA: Medial neck shaft angle
mLDFA: Mechanical lateral distal femoral angle
MPTA: Medial proximal tibial angle
JLCA: Joint line convergence angle
LPFA: Lateral proximal femoral angle
LDTA: Lateral distal tibial angle
ICC: Intraclass correlation coefficient
